# Automatic Algorithm-Aided Segmentation of Retinal Nerve Fibers Using Fundus Photographs

**DOI:** 10.3390/jimaging11090294

**Published:** 2025-08-28

**Authors:** Diego Luján Villarreal

**Affiliations:** Departamento de Mecatrónica y Biomédica, Escuela de Ingeniería y Ciencias, Instituto Tecnológico y de Estudios Superiores de Monterrey, Monterrey 64700, Mexico; diego.lujan@tec.mx

**Keywords:** image processing segmentation, optic pathways, visual pathways, retinal nerve fiber layer, retinal nerve fiber trajectory, course of axons

## Abstract

This work presents an image processing algorithm for the segmentation of the personalized mapping of retinal nerve fiber layer (RNFL) bundle trajectories in the human retina. To segment RNFL bundles, preprocessing steps were used for noise reduction and illumination correction. Blood vessels were removed. The image was fed to a maximum–minimum modulation algorithm to isolate retinal nerve fiber (RNF) segments. A modified Garway-Heath map categorizes RNF orientation, assuming designated sets of orientation angles for aligning RNFs direction. Bezier curves fit RNFs from the center of the optic disk (OD) to their corresponding end. Fundus images from five different databases (*n* = 300) were tested, with 277 healthy normal subjects and 33 classified as diabetic without any sign of diabetic retinopathy. The algorithm successfully traced fiber trajectories per fundus across all regions identified by the Garway-Heath map. The resulting trace images were compared to the Jansonius map, reaching an average efficiency of 97.44% and working well with those of low resolution. The average mean difference in orientation angles of the included images was 11.01 ± 1.25 and the average RMSE was 13.82 ± 1.55. A 24-2 visual field (VF) grid pattern was overlaid onto the fundus to relate the VF test points to the intersection of RNFL bundles and their entry angles into the OD. The mean standard deviation (95% limit) obtained 13.5° (median 14.01°), ranging from less than 1° to 28.4° for 50 out of 52 VF locations. The influence of optic parameters was explored using multiple linear regression. Average angle trajectories in the papillomacular region were significantly influenced (*p* < 0.00001) by the latitudinal optic disk position and disk–fovea angle. Given the basic biometric ground truth data (only fovea and OD centers) that is publicly accessible, the algorithm can be customized to individual eyes and distinguish fibers with accuracy by considering unique anatomical features.

## 1. Introduction

Any State-of-the-Art clinical device measuring structural damage must have a map that connects local regions on the optic disk (OD) (or the optic nerve head) to local trajectories of retinal nerve fiber layer (RNFL) bundles on the retina in order to compare the sensitivity loss of standard automatic perimetry [1]. As for glaucoma, which was estimated to affect 76 million people worldwide in 2020 [2], a topographically accurate prediction of restricted visual field loss due to a limited optic nerve head or peripapillary RNFL injury requires a thorough understanding of RNFL bundle traces, which sequentially is a requirement for developing new diagnostic techniques, such as fundus-oriented perimetry [3] and scotoma-oriented perimetry [4]. Combining perimetry and imaging in the case of glaucoma is also therapeutically useful for integrating the findings from structural and functional tests and increasing the sensitivity and specificity of illness detection and progression assessments [5]. Structure–function research is currently focused on mapping the spatial connection between retinal locations and peripapillary RNFL sectors [6]. These precise topographical localizations of the path of RNFL bundles on the retinal surface and their point of entrance into the OD are necessary for precise analyses of visual field defects brought on by localized optic nerve damage typically observed and detected via optical coherence tomography (OCT) and fundus images [1]. In particular, eye fundus images detailed as color photographs also provide valuable information about the condition of the retina and its key structures: the macula, fovea (FO), OD, vascularization, and the distribution of RNFL bundles. Similarly, imaging data could be helpful in creating customized functional tests that are specific to a patient and tailoring the areas examined during a perimetric technique [5]. Research into the composition and functioning of RNFL bundles is fundamental to understanding the visual system [6]. According to studies by Iwata et al. [7], inspecting and assessing RNFL bundles proves to be a valuable diagnostic tool for several types of optic neuropathy. Consistent with Diekmann et al. [8], axonal damage to the optic disk is the initial trigger for the pathogenesis of glaucoma, which leads to the degradation and apoptosis of retinal ganglion cells, the loss of retinal nerve fibers, and the thinning of the nerve fiber layer. Early diagnosis of glaucoma is critical, as the currently established treatment options can prevent or slow the progression of damage, but are not yet able to repair the loss of RGC axons, which results in lost connections to the central visual system [6].

Numerous attempts have been made to describe RNFL bundle trajectories, and several different techniques applied to patient populations to relate OD and RNFL traces (or visual field locations) have been published. Garway-Heath et al. [9] traced the detectable RNFL bundles of 69 eyes from the OD to visual field points on a 24-2 grid pattern overlaid on fundus photographs. Lamparter et al. [10] traced the RNFL bundle distribution of 100 eyes by setting a minimum of three marker points and connecting them using a nonparametric cubic spline model, and providing the impact of ocular parameters on the mapping of retinal locations to the OD. In a similar manner, Jansonius et al. [11] traced the visible RNFL bundles of 55 eyes in fundus photos and then fitted a mathematical model to the data, enabling extrapolation to additional central retinal regions. Denniss et al. [5] recently developed a simulation model of the association between OD sectors and visual field point locations for a range of clinically plausible anatomical parameters. While time-consuming and subjective, manual tracing by experts on fundus photographs or OCT images serves as a “ground truth” or a baseline for comparison. Yet, a significant distinction between an automatic generated model and those currently published represents significant potential for betterment. The ground truth models necessitate either very costly ophthalmic equipment to obtain some of the required input data [5,12] or excessively long man-hours of labor and time-consuming procedures to manually [9,10] or electronically [11,13] trace RNFL bundles as far as a person can see. Therefore, it is required to generate a publicly available automated model that generates personalized RNFL bundle maps of individual eyes with high precision using publicly available inputs.

Recent advances in artificial intelligence have demonstrated how deep learning algorithms can be used to automatically learn task-specific features from high-dimensional data in a variety of medical domains and model intricate non-linear relationships. The primary benefit of the deep learning algorithm is that it is an end-to-end learning algorithm, meaning that rather than requiring a specific mechanism to tackle complicated issues, it learns these mechanisms during training. Zhiqi et al. [14] developed a deep learning model to estimate pointwise functional outcomes directly from segmentation-free 3D OCT volumes and compared the performance with the model trained with segmentation-dependent 2D OCT thickness maps. Park et al. [15] developed a deep learning architecture according to Inception V3 to predict visual fields using OCT imaging. A convolutional neural network architecture was constructed to predict visual fields using the combination of the acquired two OCT images, the macular ganglion cell–inner plexiform layer and peripapillary retinal nerve fiber layer thicknesses. Globally (the entire visual field area), for all patients, the root mean square error (RMSE) between the actual and predicted visual fields was 4.79 ± 2.56 dB, with 3.27 dB and 5.27 dB for the normal and glaucoma groups, respectively. Christopher et al. [16] developed a deep learning architecture to predict glaucomatous visual fields from OCT images. ResNet was employed as the deep learning architecture for predicting visual field global indices. Several SD-OCT OD images (RNFL thickness maps, RNFL en-face images, and confocal scanning laser ophthalmoscopic images) were inputted and the predictions for each type were compared. The best mean absolute errors between the real and predicted values were 2.5 dB (mean deviation) and 1.5 dB (standard deviation). To better understand how neural systems function and provide measurements for glaucoma progression analysis [17], it is crucial to gather the most relevant anatomical, physiological, and pathological data available about the nervous system’s structure. Similarly, an algorithmic methodology can help to direct efforts towards generating RNFL tracings using computerized models [1,5,9,10,11,12,13,18,19,20,21,22], and also towards diagnosing RNFL defects by analyzing fundus images using image processing techniques [23]. Despite the advances, artificial intelligence’s efforts exclude the segmentation of RNFL bundle trajectories, which is crucial for a topographically accurate prediction of the restricted visual field loss [3], the reach specificity of illness detection and progression assessments [5], the localization of damage and the structure–function correlation [9], and the quantitative assessment and monitoring of disease progression [23]. Deep learning training models have still not yet fully or robustly solved the problem of RNFL bundle segmentation. Several reasons for this include the variability in image acquisition protocols and database characteristics (e.g., resolution, patient demographics). Narrow population data reduces group accuracy, and retinal morphology variations across populations can amplify this effect, leading to disparities in disease results [24]. Distinguishing between histologically distinct sub-layers is challenging for deep learning models because of the reduced resolution of deeper tissues caused by the light intensity decreasing with depth. Similarly, segmentation methods can be complicated by speckle noise, which is a natural feature of OCT images [25]. 

The purpose of this work is to develop an algorithm for the segmentation of personalized mapping estimates of RNFL bundle tracings using image processing techniques. The algorithm considers personalized anatomical parameters that can be customized to individual eyes using input ground truth data of the FO and OD centers. The algorithm was verified via two different tests. In the first test, (1) a quantitative comparison of the pointwise angles of orientation between the Jansonius map and the algorithm was computed using five distinct fundus image datasets. This local angular difference tests the performance of the curvature-related analysis of the RNFL bundles in the algorithm. The difference in the pointwise angles of orientation was evaluated using the root mean square error, the mean, and the standard deviation. In the second test, (2) a quantitative comparison of the entry angles of RNFL bundles into the OD was carried out between Garway-Heath et al. [9], Lamparter et al. [10], Jansonius et al. [13], and the algorithm. This quantitative comparison tests the performance efficacy of the RNFL bundles’ convergence at the OD by quantitatively assessing the topographical relationship between particular OD regions and the associated retinal RNFL bundles. The RMSE values of the average mean entry angles of [9,10,13] and that of the algorithm were also computed. Possible bias and variation were inspected between individual maps according to personalized anatomy. Visible nerve fiber bundle tracings found using the algorithm were the foundation for comparing bias and variation with previous studies. The computational algorithm provides robust results and holds the advantage that any given combination of anatomical parameters can produce individual maps, involving those that are uncommon and so unlikely to occur in small-scale population research.

## 2. Materials and Methods

### 2.1. Subjects and Fundus Photographs

Digitized fundus images of 300 eyes of 300 subjects (277 healthy individuals without any sign of a history of ocular diseases and 33 diabetic subjects without any sign of diabetic retinopathy) were collected from five different databases: the High-Resolution Fundus (HRF) [26], Joint Shantou International Eye Centre (JSIEC) [27], Retina Identification Database (RIDB) [28], DRIVE [29], and MESSIDOR [30]. According to [31], diabetes does not affect the trajectories or their visibility as long as there is no retinopathy. The HRF (Friedrich-Alexander University Erlangen—Nuremberg) dataset consists of 45 8-bit RGB fundus photographs of 2336 × 3504 pixels using a Canon CR-1 fundus camera (Canon Inc., Tokyo, Japan) in a 45° field setting. A total of 15 images were extracted from healthy patients. The JSIEC dataset (collected between September 2009 and December 2018) consists of 8-bit RGB fundus images of 3046 × 2572 pixels using a ZEISS FF450 Plus IR Fundus Camera (Carl Zeiss Meditec AG, Jena, Germany) (2009–2013) and Topcon TRC-50DX Mydriatic Retinal Camera (Topcon Corporation, Tokyo, Japan) (2013–2018) in a 35–50° field setting. A total of 38 images were extracted from healthy patients. The RIDB dataset consists of 100 8-bit RGB healthy retinal fundus images (individuals with no retinal disease or anomalies) of 1504 × 1000 pixels, captured using a TOPCON-TRC-50 EX (Topcon Corporation, Tokyo, Japan) Fundoscope in a 45° field setting. A total of 100 images were extracted from healthy patients. The DRIVE database consists of 400 8-bit RGB fundus images of 768 × 584 pixels, captured using a Canon CR5 non-mydriatic 3CCD camera (Canon Inc., Tokyo, Japan) in a 45° field of view. The screening population consisted of 400 diabetic subjects between 25 and 90 years of age. A total of 33 photographs were selected since they did not show any sign of diabetic retinopathy. The MESSIDOR database consists of 1200 8-bit RGB fundus images of 1440 × 960, 2240 × 1488, or 2304 × 1536 pixels, captured using a color video 3CCD camera mounted on a Topcon TRC NW6 (Topcon Corporation, Tokyo, Japan) non-mydriatic retinograph in a 45° field of view. An excel file is provided, with medical diagnoses for each image. Care was taken to select 114 healthy fundus images. The image database collections are thought to comply with all relevant ethical regulations for studies involving human subjects and research protocols, as approved by the corresponding Ethics Committee.

### 2.2. Image Preprocessing

Fundus images were preprocessed in order to obtain the binary mask field of view (FOV). Unwanted text images such as patient information and neighboring low pixel noise were removed by determining the binary mask FOV, obtained by—in the following order—filtering the fundus using Prewitt operator kernels, image dilation of size nxn with structuring element neighborhoods of 1, thresholding low-pixel areas, and computing the convex hull with eight connected neighborhoods. The binary mask extracted the FOV region of interest (ROI) in the entire image.

### 2.3. Segmentation of Personalized Mapping Algorithm

Removing blood vessels is essential for accurately detecting RNFL defects and distinguishing the orientation of RNFLs from that of the vessels. Before eliminating the vessels, two separate procedures were performed for reducing noise and illumination correction using the green channel of the fundus image. The green channel maximizes the contrast between blood vessels, RNFL bundles, and neighboring regions, indicating a bright image with high contrast amongst its blue and red channel competitors [32]. For noise reduction, a median filter was applied to the masked image, replacing the pixel value with the median of its neighbors. For correcting illumination, a sigmoid curve function (or S-shaped curve) was used as a non-linear mapping function to adjust the intensity of pixels and improve low-light images. The S-shaped mid-tone section (or the middle of the curve where the slope is at its steepest) stretches the intermediate tones of the original image, making them brighter and increasing their contrast to reveal textures and features that were previously hidden. Several techniques have been reported for blood vessel extraction from fundus images [33,34]. In the current study, blood vessels were removed by applying contrast-limited adaptive histogram equalization (CAHE) [35] for contrast enhancement and dilating the resultant of enhancing elongated structures using a Hessian-based multiscale Frangi vesselness (HFV) filter [36]. Achieving better results involves “cleansing” the image by applying thresholding to low-pixel areas. CAHE amplifies contrast until the output region’s histogram roughly resembles a distributed curve histogram. Contrast enhancement is limited to prevent enhancing any noise that may be present in the image. An alternative algorithm was employed for optimizing the filter’s response by estimating the thickness of the tubular structures present in the fundus. HFV filtering is a prominent case of a feature enhancement filter, particularly for the segmentation and analysis of tubular structures. The elements of the Hessian matrix, also known as second-order local derivatives, quantify the local curvature of the intensity landscape. The elements capture how the gradient of the image intensity changes, which is crucial for identifying distinct geometric patterns [36]. A complementary algorithm preprocesses the green additive primary color of the RGB fundus for retinal nerve fiber (RNF) segmentation via—in the following order—box–kernel low-pass filtering, a modified CAHE algorithm for contrast boosting, and sigmoid function intensity transformation for high-contrast stretching. The preprocessed monochromatic image is fed into a maximum–minimum modulation (MM) algorithm that isolates RNF segments from the surroundings. The MM algorithm redistributes pixel intensities through intensity normalization, which increases the luminance difference between the target segment and its surroundings. As a result, the segment’s borders are more salient for later edge detection and thresholding. The resulting monochromatic image and blood vessel extraction are binarized using the Otsu method, followed by image subtraction. Multiple algorithms for RNF alignment are executed as follows. Unless otherwise stated, RNFs described by their centroid and the angle of orientation are known as “pointwise RNF extraction”. A modified Garway-Heath map [9] classifies the pointwise RNF orientation, assuming expected sets of angles seen from the OD with high variance for adjusting the extractions across several areas. The sets of angles were based on the direction angles per sample point of the Jansonius map seen from the OD center. The direction angles were relocated to a pixel image, placing the FO in the image center. Image dilation was carried out to avoid pixel spacing. Each area was superimposed, and the set of angles was determined by obtaining the maximum and minimum of the angles within the area. The classification used the criterion based on the pointwise RNF angle; any angle falling outside the defined set was excluded from further analysis. This criterion eliminates false positive cases by highlighting non-RNFL bundle structures. For the complete process, see Validation Procedure. A complementary algorithm incorporates “group” and “individual” averages to ensure the continuity of orientation between neighboring extractions. An interconnected algorithm assembles RNFs by identifying and connecting the optimal nearest pointwise extractions based on the orientation angle. The 4°-order Bezier curve function fitted the assembled RNFs. Key representative points were chosen for the fitting procedure, including the OD center, the final, and the highest point. Bezier curves were selected for a homogeneous solution, including the fitting of RNFL bundles that fail the vertical line test (vertical line intersects the curve more than once), typically seen entering the inferonasal sector of the OD and thus presenting significant challenges for fitting procedures. This singularity was observed in Figure 2a in [11], Figure 3 in [13] and Figure 4a,b in [31]. The curves are traced from the center of the OD to their corresponding end. At least five pointwise RNF extractions are required for fitting a 4°-order Bezier curve. The spatial trajectories from local regions on the retina are connected to the OD by intersecting individual bundle maps with the OD boundary to determine the entry OD angle locations. A standard value of 60 degrees was used, since several databases omit the OCT field of view (visual angle).

A MATLAB (MathWorks, Inc., United States, Version 9.14) script and Image Processing Toolbox^TM^ were used to develop and implement the algorithm for the segmentation of personalized estimate mapping. For accessibility, the PES (personalized estimated segmentation) software application was built using a graphical user interface (GUI) in MATLAB by an app designer that offers interactive, user-friendly, and straightforward tools to generate nerve fiber estimate trajectories. The application is ready-to-download and can be used in MALTAB (see Data Availability Statement).

### 2.4. Ocular Parameters

The influence of the following ocular parameters on the average RNF orientation angle was investigated. The binary mask field of view obtained before calculated the FOV size, known as the radius at the retina (or one half of the mask in the horizontal line). Two algorithms separately enhanced the FO ROI and two algorithms for the OD ROI in order to obtain different results by specifically targeting contour edges for accurate identification. Consideration was first given to excluding fundus images for cases when the FO and/or the OD could not be located precisely. Concerning the assumption of the OD center, the clearest image representation of the OD ROI contour from the two algorithms was chosen to select five recognizable points around the OD boundary, and the best fit for an elliptical-shaped closed contour was computed using the Gal [37] algorithm using the fit estimation method of the Least Squares method. The OD center and horizontal and vertical OD radii were computed. Despite the Gal algorithm being able to identify rotated elliptical shapes, the OD tilt was not used as an independent variable since Spectralis OCT validation is still required. Concerning the FO center, the clearest image representation of the FO ROI contour was chosen to select its center. The position of the OD in relation to the FO is assumed as the horizontal distance between the OD center and FO ($OD_{x}$), and the vertical distance between the OD center and the horizontal meridian ($OD_{y}$). Distances were measured in degrees. The ellipticity ratio is assumed to be the ratio between the shortest and largest OD radius. The disk–fovea angle is assumed to be the inverse function of the tangent of the $OD_{y}$ to $OD_{x}$ ratio, measured in degrees. The OD area is assumed to be the product of the horizontal and vertical OD radii and π, measured in degrees.

### 2.5. Validation Procedure

#### 2.5.1. Pointwise Validation (1st Test)

The FO and OD center positions were marked on each fundus image. As recently described by Jansonius et al. [11,13], each traced image was superimposed via translation for centering the FO, followed by zooming and rotation for aligning the OD centers at an eccentricity of 15°, 2° above the horizontal meridian. The resulting traces were compared to that of the Jansonius map [11,13] via the following procedure. The Jansonius map was traced with a 1-degree step, and the orientation angles per sample point were calculated as seen from the OD center, centered at (15°, 2°). An algorithm identifies the sample point within the dataset closest to a specific RNF centroid and assigns the orientation angle to that of the Jansonius map. The model’s performance was optimized through iterative adjustment of the limits for each set of angles using coarse sampling interval for computational efficiency. This provides fine-tuning for each set. The percentage of effectiveness was computed for each extraction as follows:

(1) $$P_{eff}=100\left(1-\frac{\left|a_{i}-{\hat{a}}_{i}\right|}{2\pi }\right)$$

where $a_{i}$ is the corresponding angle in the dataset closest to a specific RNF extraction for the *i*th point and ${\hat{a}}_{i}$ is the orientation angle for RNF extraction in units of radians. The set yielding the highest average percentage was recorded. A total of 150 out of 300 fundus images were used to fit the nerve fiber estimate trajectories. The remaining 150 images were reserved as a test sample for the independent validation of the model. This quantitative comparison evaluated the performance of the curvature-related analysis of the RNFL bundles in the algorithm. *b* and *c* values used in the Jansonius map corresponded to the 95% limit central range covering 95% of the corresponding fits included (see Figures 3 and 5 in [11], and Figures 5 and 6 in [13]). The root mean square error (RMSE) is computed as

(2) $$RMSE=\sqrt{\frac{1}{n}\sum_{i=1}^{n}{\left(a_{i}-{\hat{a}}_{i}\right)}^{2}}$$

where n is the number of sampled data points in the RNFL bundles. The mean and standard deviation of the difference, i.e., $\left|a_{i}-{\hat{a}}_{i}\right|$, was also computed. 

#### 2.5.2. Structure–Function Mapping (2nd Test)

A properly scaled Humphrey Field Analyzer of a 24-2 visual field–test grid pattern (test points in a grid 6° apart) was superimposed onto the fundus image, centered in the FO. Only the MESSIDOR fundus image database (114 healthy, nondiabetic, without any sign of diabetic retinopathy) was used, since all images show the entire OCT field of view area with high resolution. The position of the OD in relation to the FO was recorded, and the entry points of RNFL bundles into the OD were related to all visual field locations. The entry angle of each bundle was determined by intersecting individual bundle maps with the OD boundary. The nasal margin (3 o’clock position, right eye) was designated 0°, and degrees were counted in a clockwise direction. Each RNFL bundle had to be within 0.86° (twice the diameter of the Goldman Size III stimulus) of a visual field point. To account for the inverse relationship between retinal location and visual field sites, the superior visual field places were assigned the OD entry location of their mirror image position in the inferior hemifield, and vice versa for inferior visual field locations. After assigning the entrance angles, the RNFL bundles were relocated to a pixel image, placing the FO to the image center, and image dilatation was carried out to avoid pixel spacing. The scaled visual field–test grid pattern was superimposed and the entry angles per visual field point were computed. This quantitative comparison tests the performance efficacy of the RNFL bundles’ convergence at the OD by quantitatively assessing the topographical relationship between particular OD regions and the associated retinal RNFL bundles. The number of pixels in the circumference of the visual sites, which is determined by the chosen image resolution, is equal to the maximum number of crossing bundles. As the OD sectors formed a circular domain (that is, sectors 1 and 360 were adjacent despite their large numerical variance), ranges of mapped OD sectors for each visual field location were examined for discontinuities around the 1/360 border. In all cases where discontinuity was higher than 180°, it was subtracted 360° from all sectors to make the range continuous. The RMSEs of the entry angles at the OD were computed as in Equation (2). n is the number of visual field points, $a_{i}$ is the average mean of the entry angles of [9,10,13], and ${\hat{a}}_{i}$ is the entry angles obtained in the algorithm.

### 2.6. Statistical Analysis

Multiple linear regression studied the associations between individual deviations by determining the influence of the following factors possibly associated with the trajectories: OD area, OD position, disk–fovea angle, ellipticity ratio, and FOV size (pixels). The outcome measure (or dependent variable) is the average RNF orientation angle (of extractions) in the papillomacular region, where noticeable sinuous deviations occur, as the curve’s apex—where the first derivative is zero, indicating a horizontal tangent line—and in some cases the inflection point can be identified along the RNFL bundle trajectory. As the RNF sectors created a circular domain with discontinuities near the adjacent borders of 1/360 and 180/-180, the algorithm evaluated all situations by subtracting 360 from any sectors less than 0 to maintain continuity for the regression and develop a convention of angles. Thus, for the inferior hemifield, the angles ranged 0° to 180°, indicating a transition from the nasal side to the temporal side. Conversely, for the superior hemifield, the angles ranged from 180° to 360°, representing a movement from the temporal side back to the nasal side. Consistent with Leung et al. [38], the papillomacular region includes the papillomacular and papillofoveal bundle regions. It is important to note that multiple lineal regression does not include normalization/rotation techniques, which decrease the between-subject variability by combining the OD and FO centers in line [10]. Multicollinearity was found via Pearson’s correlation matrix. The components with a correlation coefficient greater than 0.5 were included in the multiple linear regression analysis. Statistical significance in the regression was defined as *p* < 0.00001. All statistical analyses were carried out in Statistics and Machine Learning Toolbox^TM^ in the MATLAB environment.

## 3. Results

Of the 300 fundus images, 33 were classified as diabetic without any sign of diabetic retinopathy. The algorithm presented here successfully traced RNFL trajectories per fundus across all regions identified by the Garway-Heath map. The algorithm extracted an average of 7854 (HRF), 10,139 (JSIEC), 4539 (RIDB), 7977 (DRIVE), and 4445 (MESSIDOR) RNF centroids per fundus image and per database, for an average estimated total number of 1,732,657 (which is equal to $P_{eff}$). Fundus images and the status of the eye (healthy, glaucoma subject, or glaucoma) were not included in the algorithm, but were instead reserved for future tests according to eye status. The algorithm assembled an average of 6058 (HRF), 8334 (JSIEC), 4138 (RIDB), 5122 (DRIVE), and 3862 (MESSIDOR) RNFs per fundus image and per database, for an average estimated total number of 1,435,128. On average across all fundus images, the proposed algorithm reached 97.44% effectiveness and worked well with fundus images of low resolution. On average, the number of fiber extractions was 6990, which assembled 5502 retinal nerve fibers per fundus image. 

**Figure 1:**
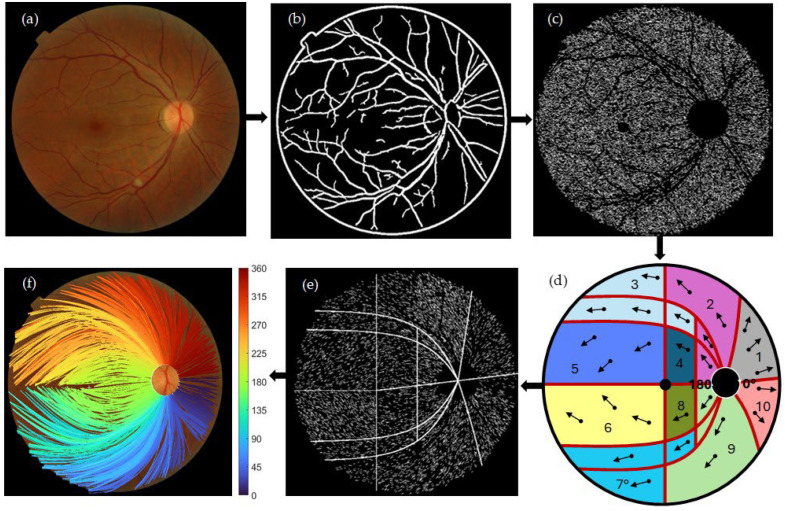
Block diagram for the segmentation of personalized mapping of the optic pathways using image processing techniques. (**a**) original RGB fundus image. (**b**) Blood vessel segmentation using CAHE and Frangi vesselness filter. (**c**) RNF segmentation via maximum–minimum modulation algorithm. (**d**) A modified Garway-Heath map [13] categorizes RNF orientation, assuming designated set of angles for aligning RNFs’ direction across various areas. The convention of angles is drawn around the OD boundary. (**e**) Pointwise RNF extractions described by their centroid and the angle of orientation seen from the center of the OD. (**f**) Assembly of RNFs by identifying and connecting the optimal nearest extraction based on the orientation angle. Bezier curves were employed to progressively fit the assembled RNFs from the center of the OD to their corresponding end. Crossing individual bundle maps with the OD boundary was used to determine the entry OD angle locations. Beside (**f**), a colormap depicting the entry OD angle sites is displayed.

Figure 1 shows the block diagram of the algorithm presented here for the segmentation of the personalized mapping of the optic pathways using image processing techniques. The RGB fundus was preprocessed by determining the binary mask field of view (FOV). Noise reduction and illumination correction were applied using a low-pass filter and a low-light enhancement sigmoid curve function. Blood vessels were removed by applying CAHE and Frangi vesselness filter algorithms, see Figure 1b. A complementary algorithm optimizes the filter’s response by estimating the thickness of the blood vessel structures across the fundus. For RNF segmentation, a complementary algorithm processes the green channel of the RGB fundus image, which is then fed in a maximum–minimum modulation algorithm to isolate RNF segments. The monochromatic image and extracted blood vessels are binarized, followed by image subtraction, see Figure 1c.

A modified Garway-Heath map categorizes RNF orientation, assuming designated sets of angles for aligning RNFs’ direction across various regions. The convention of angles is shown around the OD boundary, see Figure 1d. An additional algorithm utilizes several averages to guarantee the consistency of orientation among adjacent extractions, see Figure 1e. A connected algorithm compiles RNFs by connecting the nearest optimal extraction based on the orientation angle. Bezier curves were used to fit the assembled RNFs from the center of the OD to their corresponding end. The spatial trajectories on the retina are connected to the OD by crossing individual bundle maps with the OD boundary to determine the entry OD angle locations (shown beside colormap, see Figure 1f).

**Figure 2:**
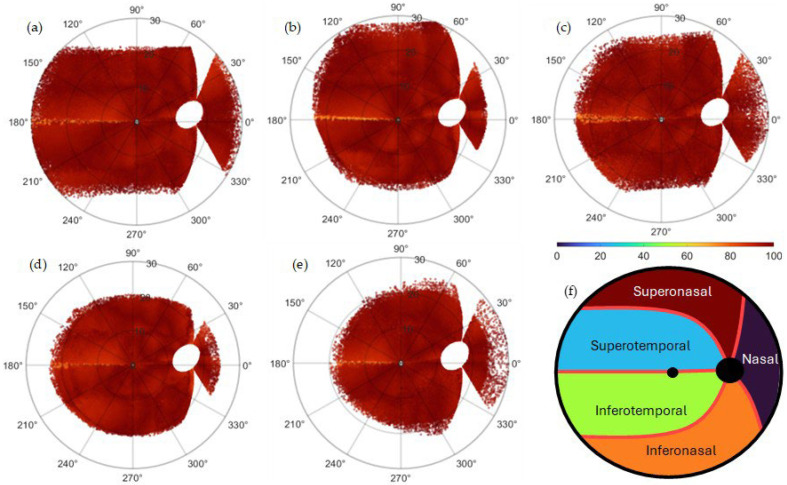
Percentage of effectiveness as part of the validation process per database: (**a**) HRF, (**b**) JSIEC, (**c**) RIDB, (**d**) DRIVE, and (**e**) MESSIDOR. The images were superimposed per database and onto the Jansonius map via translation for centering the FO, followed by zooming and rotation for aligning the OD centers at an eccentricity of 15°, 2° above the horizontal meridian. The percentage of effectiveness is computed as in Equation (1). The algorithm extracted an average of 7854 (HRF), 10139 (JSIEC), 4539 (RIDB), 7977 (DRIVE), and 4445 (MESSIDOR) RNF centroids per fundus image per database, for an average estimated total number of 1,732,657 (which correspond to the same number of $P_{eff}$). (**f**) General Garway-Heath map used to classify the results is shown in Table 1. The map is a modified version of the structure–function map developed by Garway-Heath et al. [9].

Figure 2 shows the percentage of effectiveness per database as part of the validation process. The fundus images were superimposed per database and to the Jansonius map via translation for centering the FO, followed by zooming and rotation for aligning the OD centers at 15° of eccentricity, 2° over the horizontal meridian. The percentage of effectiveness is computed as in Equation (1).

**Figure 3:**
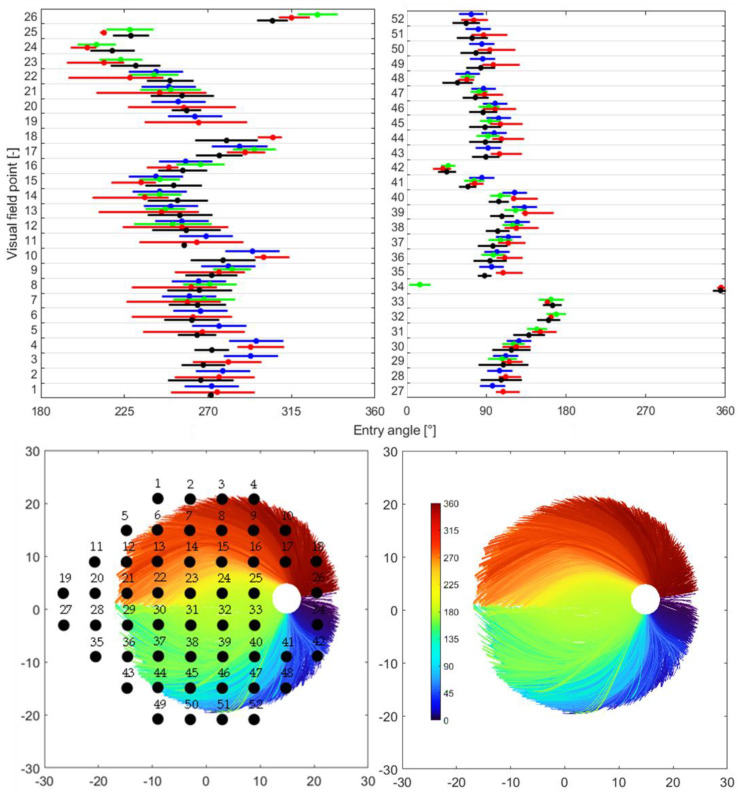
Distribution of RNFL bundles and their consistent visual field positions. At the top are the quantitative comparison of the distribution of mapped OD sectors from this study and the results published by Garway-Heath et al. [9], Lamparter et al. [10], and Jansonius et al. [13]. Each plot represents one hemifield region of the Garway-Heath et al. map [9], and each row within a plot represents the visual field sites. The blue line represents the mean standard deviation (95% limit) as reported by [9]. The red line represents the mean and upper/lower limits of the predicted OD sectors from [13]. The green line represents the mean standard deviation as reported by [10]. The black line represents the mean standard deviation (95% limit) as reported in the current study. At bottom left is the RNF bundle distribution: 34,718 RNFL bundles, based on roughly 48,930 pointwise RNF extractions, were derived from only seven subjects. A total of 52 visual field points are superimposed onto the RNFL bundle image. For reporting, the superior visual field sites are assigned to the OD entry position of their mirror image location in the inferior hemifield, and vice versa for inferior visual field site. At bottom right is the RNFL bundle distribution.

The algorithm extracted an average of 117,810 (HRF), 395,421 (JSIEC), 453,900 (RIDB), 263,241 (DRIVE), and 502,285 (MESSIDOR) RNF centroids per database. A modified Garway-Heath map [9] used to classify the results in Table 1 is shown. For validation, the frequencies (percentage) higher than 92.5 (percentage of effectiveness) were summed per region and database and shown in Table 1. The average per database is 97.22% (HRF), 94.30% (JSIEC), 97.82% (RIDB), 94.37% (DRIVE), and 97.32% (MESSIDOR). Figure 3 shows the distribution of RNFL bundles and their consistent visual field positions.

At the top are the quantitative comparison of the spreading of mapped OD sectors from this study and the outcomes reported by Garway-Heath et al. [9], Lamparter et al. [10], and Jansonius et al. [13]. Each plot corresponds to the hemifield regions (superior and inferior) of the Garway-Heath et al. map [9], and each row within corresponds to the visual field sites. The blue line represents the mean standard deviation (95% limit) as reported by Garway-Heath et al. [9]. The red line denotes the mean and upper/lower limits of the predicted OD sectors from the Jansonius map. The green line represents the mean standard deviation as published by Lamparter et al. [10]. The black line represents the mean standard deviation (95% limit) as reported in the current study. At the bottom left is the RNF bundle distribution; 34,718 RNFL bundles, based on roughly 48,930 pointwise RNF extractions, were derived from only seven subjects. A total of 52 visual field points were superimposed onto the RNFL bundle image. For reporting, the superior visual field sites were assigned to the OD entry position of their mirror image location in the inferior hemifield, and vice versa for the inferior visual field site. At the bottom right is the RNFL bundle distribution.

**Figure 4:**
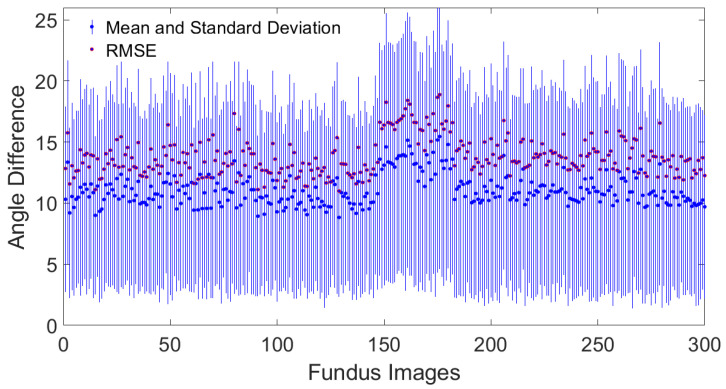
Quantitative comparison of the root mean square error, the mean, and standard deviation of the orientation difference between this study and the results reported by Jansonius et al. [13].

Figure 4 shows the quantitative comparison of the root mean square error, the mean, and the standard deviation of the differences in orientation angles between this study and the results reported in the Jansonius map.

**Figure 5:**
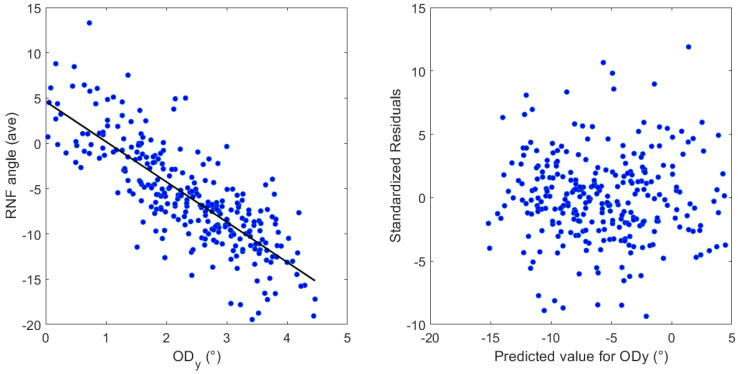
Left, the influence of the latitudinal distance of the OD center ($OD_{y}$) on the average RNF orientation angle. The average RNF orientation angle along the *y*-axis is subtracted from 180. Right, standardized residuals against the predicted value for $OD_{y}$.

Table 2 presents the results of multiple regression that show that vertical OD distance and disk–fovea angle are statistically significant on the average RNF orientation (*R*^2^ = 0.64, *β* = −4.43, *p* < 0.00001) and (*R*^2^ = 0.62, *β* = −1.56, *p* < 0.00001), respectively. Figure 5 shows (left side) the influence of $OD_{y}$ on the average RNF orientation angle in the papillomacular region. The average RNF orientation angle along the *y*-axis is subtracted from 180. On the right side, standardized residuals are shown against the expected value for $OD_{y}$. 

## 4. Discussion

This work addresses the inherent challenges of accurately segmenting RNFL bundle traces in the human retina, particularly by circumventing the limitations often encountered in deep learning-based methodologies. While deep learning models excel in automatically learning task-specific features from high-dimensional data and modeling intricate non-linear relationships [14], they can hinder their clinical applicability and interpretability in complex anatomical tasks [24,25]. In contrast, the proposed algorithm leverages advanced image processing techniques to intrinsically identify and delineate RNFL bundles, reducing dependency on vast training data and offering greater transparency in its decision-making process. A key innovation lies in the integral use of a structure–function map, which guides patient-specific bundle segmentation by incorporating known anatomical–functional correlations of the OD and its corresponding visual field. This integration not only enhances the accuracy and biological plausibility of the segmented traces, but also provides a clinically relevant framework for evaluating RNFL health, leading to more robust and interpretable results than purely data-driven, deep learning alternatives. Using a single algorithm to track RNFL bundles on the retinal surface can significantly enhance the identification of localized optic nerve damage and visual field defects. Such advances could greatly improve clinical evaluations in the diagnosis and treatment of visual disorders, including visual field loss and glaucoma.

### 4.1. Literature Review

As mentioned in several works, many other descriptions of nerve fiber tracings have been published before. Wigelius et al. [19] provided a mathematical description in his thesis that included an implicit solution similar to the trajectory path described by the Jansonius map. Nevertheless, the fitting of the trajectory density on a circle surrounding the optic disk and local RNFL thickness was not achieved. An RNFL bundle map based on scotoma borders in bundle defects was created by Weber and Ulrich [18]. This map, while less precise and comprehensive, revealed a pattern that was somewhat identical to the concept described by the Jansonius map. Numerous publications have previously presented descriptions of RNFL bundle trajectories. The publications were derived from postmortem human eyes [39], monkey histology [40], fundus photography [9], associations among optic disk anatomy and perimetric sensitivities by Ferreras et al. [20] and Turpin et al. [21], and optical coherence tomography by Garvin et al. [41]. Jansonius et al. [11,13] developed a mathematical model that explains the typical trajectory and variations in retinal nerve fiber trajectories. Leung et al. [12] presented an RNFL optical texture analysis (ROTA) algorithm, which integrates RNFL reflectance and RNFL thickness data obtained from OCT scans to uncover the optical textural details of RNFL bundles via a series of non-linear transformations. Dennis et al. [5] proposed a similar method to the one presented here, developing an anatomically customizable fiber mapping method by dividing the OD head and the retina into sectors and into a grid of elements, respectively. The amount of RGCs in each grid element was based on empirical data. The pathway from each element to the optic nerve head was determined by the number of axons that could enter the optic nerve head in each sector, as well as the sequence in which axons “grew” from different regions at the retina.

### 4.2. Concordance and Discordance with Existing RNFL Bundle Paths

The concordance of the algorithm’s output with the mathematical model proposed by Jansonius et al. [13] is in good quantitative and qualitative agreement for most locations, see Figure 2. The average mean difference in the orientation angles of the included images is 11.01 ± 1.25, and the average RMSE is 13.82 ± 1.55, see Figure 4. This concordance holds except with discordance occurring in the horizontal line at the latitude temporal to the fovea, see Figure 2. According to Jansonius et al. [31], “horizontal” is defined as parallel to the straight section temporal to the fovea, known as the location of the raphe. The discordance in the horizontal line temporal to the fovea may be explained by the fact that when tracing RNFL bundles close to the location of the raphe, it is extremely difficult to tell which fibers originate from and which pass over that point of interest. According to previous studies such as Jansonius et al. [11], all visible RNFL bundles from 55 eyes of 55 subjects were electronically traced as far as visible by one author from high-quality red-free fundus images, resulting in 1660 RNFL bundles. Jansonius et al. [31] provided another dataset of 28 fundus pictures of the right eyes of 28 subjects, including only those without diseases affecting the RNFL or its visibility (mostly diabetic patients without diabetic retinopathy). Lamparter et al. [10] manually traced RNFL bundle distribution by setting a minimum of three marker points and connecting them via a nonparametric cubic spline model, providing a continuous analytical function for each nerve bundle and obtaining 6388 RNFL bundles based on 42,914 sampling points from 100 subjects. According to those studies, RNFL bundles close to the horizontal line temporal to the fovea are neither visible nor manually drawn, excluding those crossing the line at nearly 180° minus the fovea–disk angle in the superior hemifield [10,11], and those crossing the line at nearly 180° plus the fovea–disk angle in the inferior hemifield [31]. This is consistent with the observed discordance between the algorithm presented here and the previous studies, see Figure 2. Another reason for such discordance may be attributed to the algorithmic limitations from disregarding the structural data of the optic disk, such as the tilt angle, the axial length, the spherical equivalent, and more unknowns that are not taken into consideration, which may provide hints about how to map correctly in some eyes. The architecture of the RNFL bundle may also be affected by other potential factors, such as the axial elongation that increases the OD–FO distance in myopic eyes [42], the differences in the number and distribution of ganglion cells, the layout and depth of blood vessels, and developmental growth signals [43,44]. A significant obstacle to merging imaging data with the segmentation of RNFL bundles is the significant noise that is typical of fundus images. Finally, the image resolution mostly affects contrast identification, contrast enhancement, and image filtering. Similarly, the visibility of RNFL bundles and layer defects is best recognizable in the peripapillary region and diminishes toward the periphery [45,46]. Fundus photographs provide only a two-dimensional representation of the RNFL, which is actually organized in a three-dimensional structure. The most superficial RNFL bundles are located near the vitreous body and correspond to the central region, while the deeper layers originate from the retinal periphery [47]. Radius et al. [48] and Minckler [49] demonstrated that fibers starting in the peripheral retina are located deep in the RNFL. Consistent with Garway-Heath et al. [9], the retinal nerve fibers do not seem to be highly organized within the deeper retinal layers. Consequently, fibers from the periphery, despite being visible, cannot be traced back to the optic disk [11].

### 4.3. Quantitative Performance of the Structure–Function Map

The structure–function map presented here is in good qualitative agreement with the previously published results, see Figure 3. Jansonius et al.’s [11] mean standard deviation of the angular location at the OD circumference (calculated as one quarter of the 95% limits) ranged from less than 1° to 18°, with an average value of 8.8° (median 7.3°). Lamparter et al. [10] showed a between-individual variability in the OD entry position, with a mean standard deviation of 12.4°. The variability at the level of the OD circumference found by Garway-Heath et al. [9] exhibited a mean standard deviation of 7.2°. In this work, the mean standard deviation, including a 95% limit (calculated as plus or minus two times the standard deviation), showed a value of 13.5° (median 14.01°) for 50 out of 52 visual field locations (missing only 19^h^ and 27^h^ data). Here it was shown that the standard deviation depends largely on the location of the visual field test point (from less than 1° to 28.4°). A possible explanation can be attributed to the wide range of disk–fovea angles (9.1 ± 3.9, ranged from 0.6° to 19.7°) of the recruited subjects, and similarly to the diverse variety of OD locations, which clarify the most variance in the structure–function maps [5,9]. The comparison of the current model’s output with the mathematical model proposed by Jansonius et al. [13], and the maps produced by Lamparter et al. [10] and Garway-Heath et al. [9], appears to show reasonable qualitative agreement for most locations, see Figure 2 and Figure 3. Even if the means differ numerically, all but one case (fourth visual field point) display overlapping standard deviation ranges, indicating that those groups have a common range of values because of the intrinsic variability. These findings hold potential for producing customized structure–function tests tailored to each patient and increasing the sensitivity and specificity of disease progression and detection metrics. The differences of entry angles between the average mean of [9,10,13] and that obtained here range from less than 1° to 24.9° (9.88 ± 5.95). Similarly, the RMSE had a value of 11.51. For a fair comparison, the value at the 34th visual field point is excluded because of the discontinuities around the 1/360 border.

### 4.4. Validation up to 30° of Eccentricity

An agreement has been found between the retinal wiring of the nerve fibers presented here and the principle of orderly arrangement available today [11,13,50,51], rigorously up to an eccentricity of 30°. RNFL bundles converge in an ordered fashion to the optic nerve. RNFL bundles that supply the nasal side at the superior and inferior retinas have relatively straight pathways [50,51]. This orderly assembly is visible on fibers with angular positions at the superior, inferior, and nasal edges of the OD (see Figure 1f). The trajectory starting at the OD continues in an orderly fashion by arranging fibers with consecutive angular positions, obeying the natural spatial movement. 

Those originating from the peripheral temporal retina arc positioned above or below the macular region enter the optic nerve in an arcuate pattern, into the superior and inferior temporal portions, respectively [50,51]. In this work, fibers with angular positions at the optic disk’s temporal edge are arranged in an arcuate pattern coming from the temporal hemifield in a consecutive manner. Those accommodated in the papillomacular bundle area follow arcuate patterns from the macular region toward to the optic nerve’s temporal edge.

### 4.5. Close Methodological Comparison of the Algorithm with Prior Literature

A noteworthy difference between the present algorithm and that reported previously is that the most accurate models require excessively long man-hours of work and time-consuming procedures to manually [9,10] or electronically [11,13] trace RNFL bundles as far as visible by a person, or extremely expensive ophthalmic equipment for obtaining the necessary input data [12]. Jansonius et al. [13] used high-quality fundus photograph acquisition, image processing techniques for making the fibers stand out, foveola and OD marking, image superimposition and alignment, and manual tracing with inclusion criteria as inputs. Lamparter et al. [10] used fundus photography, RNFL bundle optimization, manual delineation, and cubic splines to connect the marker points (at least three) as inputs. Dennis et al. [5] used the axial length of the eye, OD position, horizontal and vertical diameter of the OD, horizontal and vertical cup diameter, and fovea diameter. Leung et al. [12] used RNFL reflectance and RNFL thickness data obtained from OCT scans. In this work, RNFL bundles are assembled using image processing algorithms and publicly accessible inputs, see Section 2.4. The inclusion criteria were first given to exclude fundus images for cases when the FO and/or the OD could not be located precisely. Similarly, the criterion requires at least five marker points (or five pointwise RNF extractions) to fit the assembled RNFs using a 4°-order Bezier curve. The general method of precision can be attributed to the sequence of three phases. The first phase involves the CAHE for reducing undesired noise amplification while simultaneously improving local contrast, and the HFV filter for enhancing and detecting tubular patterns, which is crucial for identifying distinct curvilinear RNF structures. The second phase involves the MM algorithm, which inherently involves finding the local maximum and minimum pixel intensities within a region in order to assess edge sharpness and isolate RNFL bundle segments. Pixel intensities are redistributed, thereby improving the luminance difference between the target RNF extractions and their surroundings. The third phase aligns the orientation of RNF extractions by assuming specific sets of angles in a modified Garway-Heath map [9]. A simple criterion states that if the RNF angle falls outside the defined range for a specific area, the RNF extraction is discarded from further evaluation. This criterion eliminates false positive cases by highlighting non-RNFL bundle structures. The average range of angles for adjusting RNFs’ direction provides a broad variability (70° ± 21°). The proposed segmentation algorithm has a runtime complexity of less than 45 s for processing around 8.1 megapixels in the image (2330 × 3500), which involves a fixed number of sequential passes over the image for feature computation (including segmentation, Bezier curve fitting, structure–function mapping, and image plotting).

### 4.6. OD and FO Localization

This analysis was conducted using a diverse collection of fundus images to develop an algorithm that can be customized to individual eyes and distinguish fibers with great accuracy by considering unique anatomical features. A total of four algorithms were developed to independently boost the OD and FO ROI, focusing on contour edges for precise identification and to address problems that arise while using several databases. First, fundus images in the used collections have a broad variety of color tones surrounding the FO and OD. As a result, several of the images in this collection obscure those regions. The success rate is adversely affected, due to the fact that the OD region’s intensity is similar to that in the remainder of the fundus images. Second, there is variation in color and intensity among the images in the collection. Consequently, the methods presented here emphasize contrast illumination changes via different filtering techniques.

### 4.7. Intersubject Variability for RNFL Bundle Traces

Probable influences that could cause error bias or RNFL bundle trajectory variability are (1) the fundus photography, (2) patient selection, (3) tracing process, and (4) anatomical differences between subjects. In this work, five different fundus image databases were used, and the results revealed small variabilities from the Jansonius map’s comparison of 97.22% (HRF), 94.29% (JSIEC), 97.82% (RIDB), 94.36% (DRIVE), and 97.32% (MESSIDOR) (average per database of the summation of frequencies higher than 92.5% effectiveness), suggesting that fundus photography is neither a major source of error bias nor variability. As mentioned in the Section 2, the patients selected were healthy, without any sign of a history of ocular diseases, and diabetic subjects without any sign of diabetic retinopathy. According to [31], diabetes does not affect the trajectories or their visibility as long as there is no retinopathy. The tracing method is likewise not a significant source of variability or error bias due to the previously described similar percentage results. Concerning anatomical differences between subjects, multiple linear regression analyses show that the vertical OD distance and disk–fovea angle are statistically significant on the average RNF orientation (*R*^2^ = 0.64, *β* = −4.43, *p* < 0.00001) and (*R*^2^ = 0.62, *β* = −1.56, *p* < 0.00001), respectively. Anatomical differences between subjects have been examined before. Tanabe et al. [52] found that refraction and the architecture of the retinal arteries have significant influences on bundle angles using scanning laser ophthalmoscopy. According to Denniss et al. [5], the OD’s position in relation to the fovea, axial length, and horizontal and vertical OD diameters heavily influences the structure–function relationship. In accordance with Lamparter et al. [10], the OD’s latitudinal and longitudinal position and disk area, followed by variation in orientation and the tilt of the OD, influenced intersubject variability on entry angle into the optic nerve head. Garway-Heath et al. [9] found the influence of the OD’s position to be a parameter explaining between-subject variability. The RNFL thickness and the RNFL bundle trajectories are probably associated. As reported by Hwang et al. [53], eyes with counterclockwise rotation and a myopic temporal optic disk tilt exhibit a more temporally positioned superior peak location and a thicker temporal RNFL. According to Tong et al. [54], OD parameters and the RNFL thickness obtained with scanning laser ophthalmoscopy are strongly influenced by the tilting of the OD, but not by refractive error or axial length.

Consistent with the findings of Lamparter et al. [10], Denniss et al. [5], and Garway-Heath et al. [9], the most prominent predictor for the variations presented here is the latitudinal OD location, followed by the disk–fovea angle, which is in good agreement. Similarly, the linear regression analysis revealed robust explanatory power, with an *R*^2^ value of 0.64 and 0.62. This indicates that 64% and 62% of the variance accounted for $OD_{y}$ and the disk–fovea angle included in the model, respectively. These *R*^2^ values represent a considerable proportion of the explained variance, suggesting that the algorithm may provide a strong understanding of the factors influencing the average RNF orientation in the papillomacular region. Diagnostic analysis of the residuals showed a random distribution around zero, confirming that the assumptions of linear regression, including linearity and homoscedasticity, were adequately met. This validates the appropriateness of the linear model, reinforcing the reliability presented here, see Figure 5 (only one independent variable is shown). While the model explains a large proportion of the variance, 36% and 38% of the variability remains unexplained. This indicates that other factors, not captured in the current model, also contribute to the dependent variable. Future research could explore these unmeasured variables, such as the axial length, to further refine the understanding. Numerous studies support the concept that an increase in axial length, particularly in myopic eyes, leads to the stretching of the RNFL bundles, particularly the papillomacular bundle [55,56]. The variability in the axial length (23.4 ± 1.04 mm, range 20.29–28.68 mm [57]) imposes that any variation in its spatial relationship directly influences the length, curvature, and overall path of the papillomacular bundles [55]. This greater susceptibility results from the papillomacular bundle’s very straight trajectory, which may restrict its capacity to adjust to the growing OD–FO gap. The more peripheral nerve fibers, on the other hand, that are attached to nontemporal rim sectors, have a more arcuate path, and may thus adjust by straightening more effectively [56]. As discussed previously, one limitation imposed by the fundus images is that the axial length is not accessible and thus not used in the current study. Therefore, the clinical relevance of these significant effects awaits careful consideration.

Developing an assessment that includes the variability of the RNFL bundle’s location as a function of orientation angles could be beneficial for evaluating myopic eyes. Consistent evidence of RNFL bundle variability has been previously documented. According to several studies, the location of the peak RNFL thickness and the thickness of supratemporal and infratemporal RNFL bundles are shifted toward the fovea in eyes with longer axial lengths. This is consistent with the reports by Yoo et al. [58] and Hong et al. [59]. As the axial length increases, the RNFL bundles are likewise displaced nearer the fovea in relation to longer eyes. In a two-dimensional fundus picture, these alterations can be detected as a straightening and a shift toward the fovea of the RNFL bundles’ eyes (see Figure 1 in [60]). This RNFL bundle alteration may account for the RNFL defect frequently manifesting at the paracentral region when myopic glaucoma is first developing [60]. The RNFL bundle shift can be quantified by assessing the location of the supratemporal and infratemporal peak RNFL thickness of the peripapillary RNFL thickness profile [58]. Still, for a precise glaucoma diagnosis, it is critical to understand how myopic alterations affect the thickness of the peripapillary RNFL. However, the RNFL bundle’s displacement is not effective for the temporal raphe. Bedggood et al. [61] tested the specific hypotheses of whether myopia alters the normal trajectory of RNFL bundles temporal to the macula at the temporal raphe. The axial length had a minimal effect on the path of nerve fibers in the temporal raphe area, according to this study, but it did have some distinct effects on the thickness of papillary and peripapillary nerve fibers. Although the study conducted by Bedggood et al. [61] only examined the raphe’s shape and not the entire arc of fibers passing through the retina, the results showed that any readjustment of the fiber trajectories would not appear to include the temporal raphe (to the extent that the study was able to do so).

### 4.8. Limitations

The premise of a personalized model is that clinically measurable biometric information can improve the accuracy of mapping visual fields. This work focuses exclusively on parameters that can be determined based on research expertise such as the ground truth location data of FO and OD centers, which are publicly available. The structural data of the optic disk, such as the tilt angle, the axial length (typically assessed via partial coherence interferometry), the spherical equivalent, and the orientation of the OD (assessed via Spectralis HRA reflectance image (RNFL scan)) [10] was not considered. The fundus images used in this study were obtained from anonymized databases, and biometric data related to the axial length and refraction were not accessible. The impact of both could not be ascertained due to their absence. Accordingly, a number of factors that are not taken into consideration may provide hints about how to map correctly in some eyes. For instance, Lamparter et al. [10] found that variation in the axial length and spherical equivalent influenced the mapping of 9 out of 33 visual field sites. Deviations may also result from other possible factors influencing the architecture of the RNFL bundle, such as variations in developmental growth, the depth of blood vessels, and in the number and distribution of ganglion cells [43,44].

A significant obstacle to merging imaging data with structural maps is the significant noise that is typical of fundus image associations. There are probably several reasons for this noise, including intersubject differences in the relationship between retinal regions and the OD [5]. This topographic relationship between specific locations of the optic disk and their corresponding nerve fiber bundles on the retina is complex, and individual variation is high [9,10]. For reaching ground truth maps, experienced ophthalmologists require excessively long man-hours of labor and time-consuming procedures to manually [9,10] or electronically [11,13] trace RNFL bundles. In this work, the spatial trajectories from local regions on the retina to the OD were associated by intersecting individual bundle maps obtained with the algorithm with the OD boundary. These may provide some deviations that influence the architecture of RNFL bundles and affect the intersubject differences in the relationship between retinal regions and the OD.

The OD’s position explains the most variance in the structure–function maps, and its alteration is a significant factor affecting the RNFL bundle’s structure [5,9,10,11]. Jansonius et al. [11,13] computed the OD size from the macula–disk center distance in fundus photographs and defined the location of the OD by the papillomacular position. Lamparter et al. [10] used magnification-corrected measurements of the OD size and dislocation from the FO (in the horizontal and vertical directions, separately). In this work, two algorithms separately enhanced the FO ROI and two algorithms for the OD ROI in order to obtain different results by specifically targeting contour edges for accurate identification. However, these additional elements might affect the RNFL bundle’s design and could potentially cause deviations. Nonetheless, computational algorithms offer a useful foundation for future experimental hypothesis generation and empirical testing. The simplicity of updating and improving the algorithm in response to new empirical data can benefit the computational modeling technique. Through regular updates, this flexibility enables the algorithm to stay up to date with a rapidly evolving evidence base, allowing for more strict validation in future studies.

One recognized limitation of the HFV filter are RNF crossings and RNF bifurcations [62] as the local intensity profile at these points differs from a simple singular tubular structure and deviates from being an isolated segment with a relatively consistent intensity profile that can be characterized by its orientation (along the segment) and its cross-sectional curvature (across the segment). This limitation is alleviated by (1) aligning the RNF orientation, which eliminates false positive cases of non-RNFL bundle structures, and (2) including “group” and “individual” averages to ensure the continuity of orientation between neighboring extractions. RNFL defects in early or advanced glaucoma or other optic atrophy cannot be detected with the algorithm presented here, since it has not been tested yet. Therefore, future work should be focused on providing high reliability for the detection of optic neuropathies. The location-specific intersubject variability using the structure–function map and the overall validation using more fundus images will be addressed in future studies.

## 5. Conclusions

This work presents an image processing algorithm that segments RNFL bundle traces in the human retina. Using the FO and OD centers from fundus images as the input, the algorithm generates an accurate representation of the RNFL bundle pathways and associates each RNFL bundle with an estimated location of the OD. The quantitative comparison of the RNFL bundle traces and that of the entry angle of RNFL bundles into the OD shows three findings: (1) the segmentation of RNFL bundle tracings with great accuracy by considering unique anatomical features that can be customized to individual eyes, (2) structure–function maps can be built using image processing techniques tailored for healthy individuals, and (3) RNF orientation angles in the papillomacular region can be described using the latitudinal OD location and the disk–fovea angle. These advances could greatly improve clinical evaluations in the diagnosis and treatment of visual disorders, including glaucoma.

## Figures and Tables

**Table 1:** Frequencies (percentage) higher than 92.5 (percentage of effectiveness) per region and average per database.

Database	Nasal	Superotemporal	Inferotemporal	Inferonasal	Superonasal	Average
HRF	93.32	94.62	99.07	99.22	99.88	97.22
JSIEC	85.48	90.10	98.51	97.63	99.74	94.30
RIDB	95.77	95.29	98.41	99.86	99.80	97.82
DRIVE	87.41	89.75	97.27	97.66	99.74	94.37
MESSIDOR	97.60	90.89	98.61	99.69	99.84	97.32

**Table 2:** Multiple linear regression analyses with the average RNF orientation angle as a dependent variable and OD area, OD position, disk–fovea angle, ellipticity ratio, and FOV size as independent variables for the superior–temporal and inferior–temporal regions. All independent variables are shown, including those with a correlation coefficient lower than 0.5.

Independent Variable	Coefficient	R^2^	F-Statistic	*p*-Value
Longitudinal OD position	2.67 × 10^−1^	8.10 × 10^−3^	2.27 × 10^0^	1.32 × 10^−1^
Latitudinal OD position	−4.43 × 10^0^	6.40 × 10^−1^	4.87 × 10^2^	3.75 × 10^−63^
OD area	−2.95 × 10^−2^	3.90 × 10^−3^	1.10 × 10^0^	2.96 × 10^−1^
FOV size	−6.20 × 10^−3^	7.40 × 10^−2^	2.23 × 10^1^	3.71 × 10^−1^
Ellipticity ratio	3.16 × 10^0^	1.70 × 10^−3^	4.81 × 10^−1^	4.95 × 10^−1^
Disk–fovea angle	−1.56 × 10^0^	6.20 × 10^−1^	4.55 × 10^2^	1.42 × 10^−60^

## Data Availability

PES (personalized estimated segmentation) software application developed for the segmentation of nerve fiber trajectories is a code programmed and owned by the author written in MATLAB, available on GitHub https://github.com/diegolujv/PES-app.git (accessed on 15 August 2025).
